# Higher Media Multi-Tasking Activity Is Associated with Smaller Gray-Matter Density in the Anterior Cingulate Cortex

**DOI:** 10.1371/journal.pone.0106698

**Published:** 2014-09-24

**Authors:** Kep Kee Loh, Ryota Kanai

**Affiliations:** 1 Cognitive Neuroscience Laboratory, Duke-NUS Graduate Medical School, Singapore, Singapore; 2 Sackler Centre for Consciousness Science, University of Sussex, Brighton, United Kingdom; 3 Institute of Cognitive Neuroscience, University College London, London, United Kingdom; University of Tokyo, Japan

## Abstract

*Media multitasking*, or the concurrent consumption of multiple media forms, is increasingly prevalent in today’s society and has been associated with negative psychosocial and cognitive impacts. Individuals who engage in heavier media-multitasking are found to perform worse on cognitive control tasks and exhibit more socio-emotional difficulties. However, the neural processes associated with media multi-tasking remain unexplored. The present study investigated relationships between media multitasking activity and brain structure. Research has demonstrated that brain structure can be altered upon prolonged exposure to novel environments and experience. Thus, we expected differential engagements in media multitasking to correlate with brain structure variability. This was confirmed via Voxel-Based Morphometry (VBM) analyses: Individuals with higher Media Multitasking Index (MMI) scores had smaller gray matter density in the anterior cingulate cortex (ACC). Functional connectivity between this ACC region and the precuneus was negatively associated with MMI. Our findings suggest a possible structural correlate for the observed decreased cognitive control performance and socio-emotional regulation in heavy media-multitaskers. While the cross-sectional nature of our study does not allow us to specify the direction of causality, our results brought to light novel associations between individual media multitasking behaviors and ACC structure differences.

## Introduction

*Media multitasking*, or the concurrent consumption of multiple media forms, is increasingly prevalent in modern society [1] and has been associated with decreased cognitive control abilities [2] as well as negative psychosocial impacts such as depression and social anxiety [3], negative social well-being [4], and poor academic performance [5]. However, at this juncture, little is known about the neural processes associated with media multi-tasking. The present study investigated relationships between media multitasking activity and brain structure variability. Research has demonstrated that brain structure can be altered with prolonged exposure to novel environments [6] as well as training and experience [7], [8]. Furthermore, regional variability in grey and white matter, assessed via Voxel-Based Morphometry (VBM) reliably predicts individual differences in a range of cognitive functions (see [9] for a review). Based on the above findings, we hypothesized that differential engagements in media multitasking would likewise reflect differences in regional brain structures.

In the current investigation, the Media-Multitasking Index (MMI, [2]) is adopted as a measure of trait media-multitasking. MMI scores have been consistently associated with individual performance on cognitive control tasks [2], [10],[11]. As such, they serve as a reliable behavioral correlate with brain structure variability. We expected that an individual’s MMI score would reflect brain structure differences, specifically in cognitive control and multitasking regions. Past research has converged on the role of prefrontal cortical regions in cognitive control [12], [13], [14], [15]. Based on a lesion study by [16], distinct regions are involved in dissociable aspects of multitasking: the anterior and posterior cingulates are involved in retrospective memory, and the prefrontal regions are implicated in prospective memory and planning. As such, we expected to find associations between media multitasking activity and the structural variability in these regions. Media multitasking activity is closely linked with personality traits (i.e. neuroticism and extraversion [3]), which in turn, are predictive of structural differences in the brain [17]. As such, the association between media multitasking and brain structure might be confounded by these trait differences. To investigate this possibility, relationships between MMI and Big Five personality traits are also examined.

We obtained MMI scores, Big Five personality trait measures and magnetic resonance imaging (MRI) scans in 75 healthy adults that were relatively well-acquainted with computers and media technologies. To examine the relationship between media multitasking activity and brain structure variability, we first correlated individual MMI scores with regional gray matter density at a whole-brain level via optimized VBM [18]. We also examined correlations between the Big Five traits and MMI scores. To shed light on the functional significance of our obtained structural differences, we analyzed resting state brain activity to elucidate associations between MMI scores and functional connectivity within the brain.

## Methods

### Participants

75 healthy adults (mean age = 24.6, SD = 5.0, 38 males) recruited from the University College London (UCL) psychology participant pool took part in the current study after providing informed written consent. The study was approved by the local UCL ethics committee (ethics application code: 2213/002). We screened participants to include university students and staff that were well-acquainted with computers and media technologies. They were reimbursed in cash for their participation. Among the 75 participants who took part in the VBM study, fMRI data were collected from a subset of 40 participants. Gender, age, education level and MMI scores did not differ significantly between the two samples (Table 1).

**Table 1:** Comparisons between demographic characteristics and MMI scores of participants involved in VBM analyses and functional connectivity analyses.

Variable	VBM	Functional Connectivity	Statistic
n	75	40	
Gender (male)	38	21	χ^2^ = 0.04, *df* = 1, *p* = 0.85.
Age (years)	24.56 (5.00)^a^	24.45 (5.03)^a^	*t* = 0.11, *df* = 113, *p* = 0.91.
Highest Attained Education			χ^2^ = 0.54, *df* = 2, *p* = 0.76.
* PhD, Masters Degree*	26	16	
* Bachelors Degree*	32	17	
* High School*	17	7	
MMI Score	3.66 (1.58)^a^	3.61 (1.72)^a^	*t* = 0.16, *df* = 113, *p* = 0.88.

a Mean (standard deviation) for age and MMI scores.

### Modified Media Multitasking Questionnaire

A modified version of the Media Multi-tasking Questionnaire [2] was administered to all participants. The MMI provided a stable measure of an individual’s trait media multi-tasking activity. The questionnaire consisted of two main sections: The first section listed 12 common media types and participants reported the total number of hours per week they spent using each medium. In the modified version used in the present study, 10 media types were retained from [2]: Print media, television, computer-based video, music, voice calls using mobile or telephone, instant messaging, Short Messaging Service (SMS) messaging, Email, web surfing and other computer-based applications. The item “video or computer games” was modified to include games on mobile phones. The item “non-music audio” was replaced with “using social networking sites”. The changes were made to better reflect current trends in media consumption. The second section consisted of a matrix that involved participants indicating how much they concurrently used all the other types of medium as they used a primary medium. Amount of concurrent use was indicated on a scale of 1 to 4 (1 =  “Never”, 2 =  “A little of the time”, 3 =  “Some of the time” and 4 =  “Most of the time”). The participants’ responses were first recoded as follows: “Never”  = 0, “A little of the time”  = 0.33, “Some of the time”  = 0.67 and “Most of the time”  = 1. Summation of the recoded responses for each primary medium yielded the mean number of media concurrently used when using a primary medium. The MMI was calculated based on the following formula:
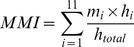
Where m_i_ is the mean number of media concurrently used while using primary medium, i; h_i_ is the number of hours per week spent using the primary medium, i; and h_total_ is the total number of hours per week spent using all media forms.

### Big Five Inventory

The Big Five Inventory (BFI; [19]) provided a brief and reliable 44-item measure for the Big Five personality factors: extraversion (8 items), agreeableness (9 items), conscientiousness (9 items), neuroticism (8 items) and openness to experience (10 items). We adopted the BFI to examine associations between MMI and Big Five personality traits in our sample.

### MRI Data Acquisition

A 1.5 T Siemens Avanto scanner (Siemens Medical, Erlangen, Germany) was used to acquire high-resolution T1-weighted structural images for each participant (MPRAGE; 1 mm^3^ cubic voxels; 160 slices; TR = 2730 ms; TE = 3.57 ms). Functional MRI data were acquired using ascending T2*-weighted gradient-echo echo-planar imaging (EPI) sequences sensitive to BOLD contrast. Each acquisition consisted of 32 oblique slices, 3.0×3.0 mm resolution, 2.0 mm in thickness with 1.0 mm slice gap. The EPI slices were angled individually for each subject to reduce susceptibility artifacts from the nasal cavity and to maximize coverage for the orbitofrontal regions and posterior parietal cortex, while sacrificing the coverage over the temporal pole. As such the final orientation ranged between 8° to 16°. The time interval between two successive acquisitions of the same slice was 2528 ms with a flip angle of 90 degrees and a 44 ms echo time. The field of view was 192×192 mm. The digital in-plane resolution was 64×64 pixels with a pixel dimension of 3.0×3.0 mm. All data were acquired with a 32-channel head coil. During the functional MRI scan, participants were instructed to simply keep still, keep eyes open, and not think about anything in particular. One run consisted of 180 volume acquisitions and the initial 6 volumes were discarded from the analysis to avoid confound of unsteady magnetization. The resting state fMRI run took approximately 7.5 minutes.

### Voxel-Based Morphometry (VBM) Analysis

Voxel-based morphometry (VBM; [20]) is a commonly used neuroimaging analysis technique that enables voxel-wise statistical analyses of pre-processed MRI images. The high-resolution T1-weighted structural scans were analyzed with VBM via Statistical Parametric Mapping (SPM8, Wellcome Department of Cognitive Neurology). The images were first segmented for grey and white matter. Diffeomorphic Anatomical Registration Through Exponentiated Lie Algebra (DARTEL) was subsequently performed to co-register the grey matter images. To ensure that regional grey matter volume was maintained after the registration, registered images were modulated by the Jacobian determinant of the flow fields computed by DARTEL. The registered grey matter images were smoothed with a Gaussian kernel (full width at half maximum = 10 mm) and were then transformed and normalized to Montreal Neurological Institute (MNI) stereotactic space for further multiple regression analysis.

Multiple regression analysis was performed on the normalized grey matter images with MMI scores as the main regressor. Age, gender and total brain volumes were included as covariates of no interest for all the regressions. To detect voxels in which regional gray matter density was correlated with MMI scores, we adopted a stringent threshold of *p<.05* with family-wise error whole-brain corrected.

### Functional Connectivity Analysis

To perform the functional connectivity analysis, we used the Conn functional connectivity toolbox version 13 (http://www.nitrc.org/projects/conn; [21]) combined with preprocessing procedures of SPM8. The preprocessing steps, listed in order, included correction for slice timing, realignment of the time series data to the first volume (i.e. motion correction), co-registration of functional MRI time series to the corresponding structural MRI, segmentation of images into separate tissue types such as grey matter, white matter and cerebrospinal fluid (CSF), and normalization to the standard MNI template and spatial smoothing with a Gaussian filter (FWHM = 8 mm). The time series data were then bandpass filtered to 0.01 Hz–0.1 Hz.

For the seed-based functional connectivity analysis, we used a single significant cluster found in the VBM analysis as the seed region-of-interest (ROI). The mean time series extracted from the ROI was used a regressor in a multiple regression model at an individual level analysis. To minimize the influences of confounding factors, regressors for the six motion correction parameters from the preprocessing were included. In addition, the mean BOLD signals for the grey matter, white matter and CSF were extracted from the masks created from the segmentation procedure, and were also included as regressors to minimize variances associated with these global signals. Temporal correlations between the ROI signal and the rest of the brain were computed and the correlations with the seed ROI were converted to Z scores using the Fisher transformation for second-level significance analyses.

With the Z-transformed statistical image, we first determined brain regions showing functional connectivity with the seed ROI using a voxel-wise threshold of *p_FWE-corrected_*<0.05. Subsequently, we used a less stringent threshold of *p*<0.001 (uncorrected) as a mask to capture ACC connected regions for a second level analysis in which we aimed to find brain regions with correlated with MMI scores. We included age, gender and total intracranial volumes as covariates and adopted a threshold of *p<0.05* with family-wise error corrected for the volume defined by the initial mask. The rationale for initial masking was to ensure that our analysis was constrained to brain regions showing correlated activity with the seed region. Even if we found a correlation with individual differences outside these regions, such findings would likely reflect spurious correlations. We adopted a less stringent threshold for the masking in order to increase the power of our second level analyses.

Processed imaging data as well as datasets containing the variables for both VBM and functional connectivity regression analyses are made publicly available at: http://dx.doi.org/10.6084/m9.figshare.1030286.

## Results

**Figure 1:**
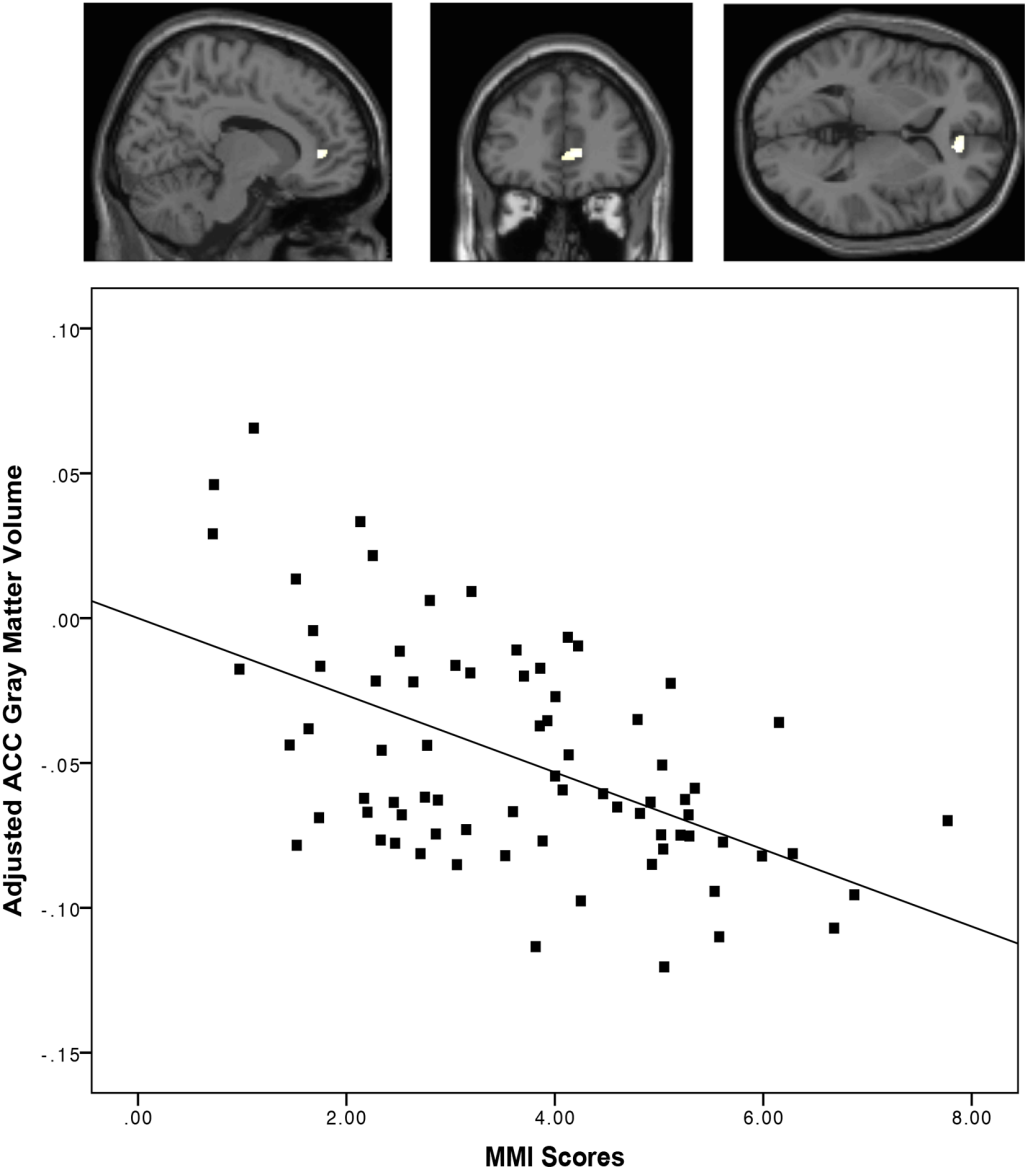
VBM regression analyses revealed that MMI scores were significantly associated with gray matter density in the ACC (t(70) = 5.16, P_FWE-corrected_ <0.05, Cluster size = 158 voxels x 1.5^3^ = 533 mm^3^; peak MNI coordinate: x = 12, y = 41, z = 3). Adjusted gray matter density in the peak voxel (Y-axis) was negatively correlated (*r* = −0.54, *p*<0.001) with MMI scores (X-axis).

VBM analysis revealed a negative association between MMI scores and gray matter density in the anterior cingulate cortex (Figure 1; ACC; t(70) = 5.16, P_FWE-corrected_ <.05, Cluster size = 158 voxels × 1.5^3^ = 533 mm^3^; peak MNI coordinate: x = 12, y = 41, z = 3). No other brain regions showed significant correlations with MMI scores. Thus, higher media-multitasking was associated with smaller gray matter volumes in the ACC. However, correlational analyses between MMI and BFI scores revealed a highly significant association between Extraversion and MMI scores (Table 2; r = 0.347, *p* = 0.002). As such, we suspected that the observed MMI-ACC gray matter association could be confounded by individual differences in extraversion scores. In view of this, we repeated the earlier VBM analysis further controlling for BFI scores as additional covariates. We ran a multiple regression (with gray matter density as dependent variable) including MMI and all Big Five trait scores as predictors along with the demographic covariates. A significant negative relationship was observed between MMI and gray matter volume in the identical ACC region (t(65) = 5.08, P_FWE-corrected_<.05, Cluster size = 74 voxels×1.5^3^ = 250 mm^3^; peak MNI coordinate: x = 12, y = 40, z = 3). This suggested that there is a unique association between MMI and gray matter density in the ACC independent of variations in the Big Five personality traits.

**Table 2:** Correlations between Media multitasking index scores and Big Five Inventory scores.

Big Five Traits	Pearson’s r	*p*
Extraversion	**0.347**	**0.002**
Agreeableness	0.173	0.138
Conscientiousness	0.070	0.552
Neuroticism	−0.131	0.262
Openness	0.100	0.391

**Figure 2:**
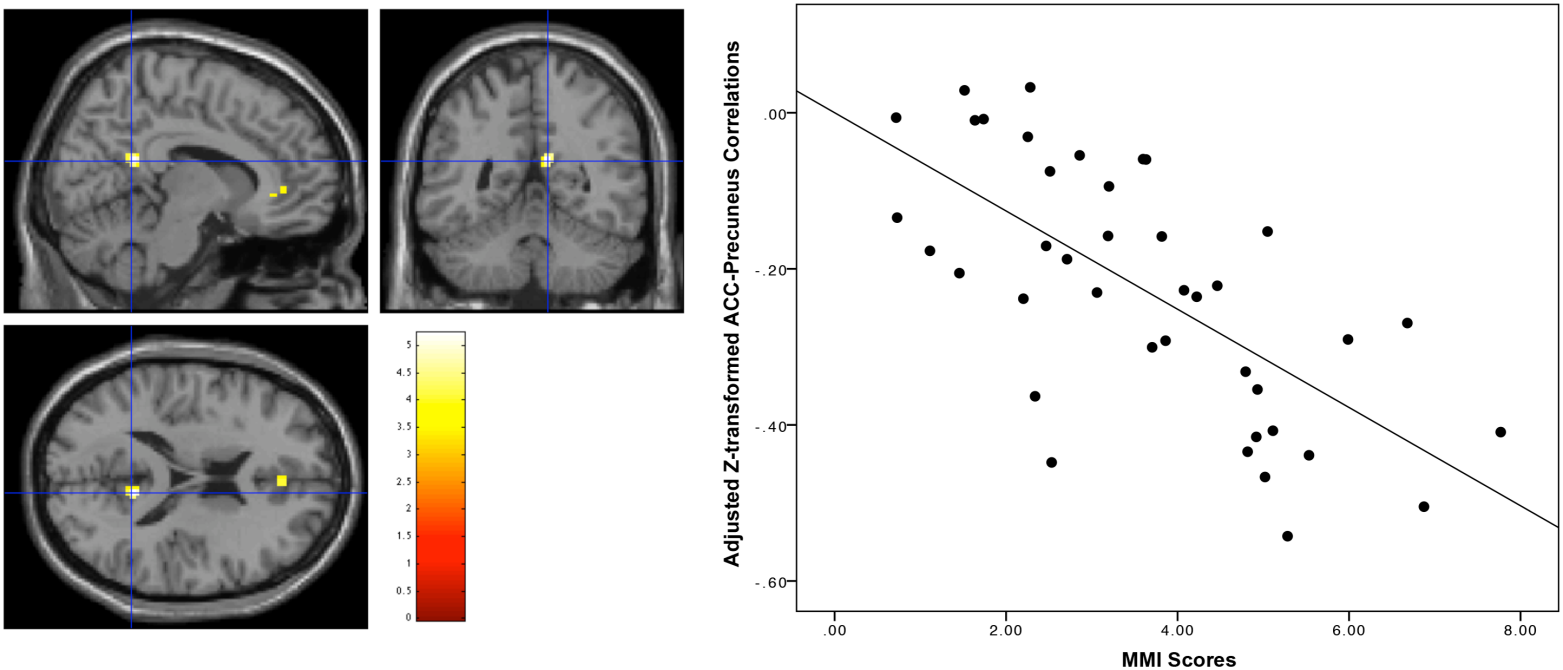
Regression analyses revealed that connectivity between the ACC ROI and the Precuneus (intersection of blue lines) was negatively associated with MMI scores (Precuneus; t(40) = 5.22, P_FWE-uncorrected_<0.001, Cluster size = 159 mm^3^; Peak MNI coordinate: x = 10, y = −50, z = 18). There was a negative relationship (*r* = −0.68, *p*<0.001) between adjusted Z-transformed ACC-Precuneus correlations (Y-axis) and MMI scores (X-axis).

To shed light on the functional significance of our VBM results, we sought to pinpoint, via functional connectivity analyses, brain regions that exhibited significant connectivity with our obtained ACC region-of-interest (ROI). This analysis revealed that activity in the obtained ACC ROI was correlated with multiple brain regions typically characterized as the Default Mode Network including the bilateral temporo-parietal junctions (TPJ; right hemisphere, x = 48, y = −64, z = 36, *p*_FWE-corrected_<0.05; left hemisphere, x = −44, y = −70, z  = 36) and precuneus (x = 4, y = −68, z = 30, *p*_FWE-corrected_<0.05) among other regions (Table 3). These results suggested that the ACC ROI we found with the VBM analysis are likely to fall within the DMN. Next, we further investigated if MMI scores were associated with connectivity between our ACC ROI and DMN regions. Regression analyses were run on z-transformed correlations between ACC and DMN regions with MMI as the main predictor and age, gender and total brain volume as covariates. No significant associations emerged at *p_FWE-corrected_<*0.05. However, at a less stringent threshold of *p_uncorrected_<*0.001, higher MMI scores was associated with weaker connectivity between the ACC ROI and the precuneus (Figure 2; precuneus; t(40) = 5.22, *p*_uncorrected_<0.001, Cluster size = 159 mm^3^; Peak MNI coordinate: x = 10, y = −50, z = 18). We emphasize that our connectivity results were obtained at a less stringent threshold and provided limited evidence for us to make conclusions about MMI and functional connectivity associations. As such, this set of findings served solely in the functional interpretation of our VBM results.

**Table 3:** Brain regions exhibiting functional connectivity with ACC ROI.

Brain Region	Brodmann’s Area		Peak Voxel		Cluster Size (Voxels)
			MNI: *x,y,z*	*t*	
R. Mid Cingulate Cortex	23	6, −28, 40		13.68	5011
R. Precuneus	7		4, −68, 39	12.12	
L. Superior Frontal Gyrus	32	−18, 28, 46		11.20	555
R. Temporoparietal Junction	39	48, −64, 36		9.70	755
L. Temporoparietal Junction	39	−44, −70, 36		9.57	609
L. Insula	48	−30, 14, −12		8.28	507

*p_FWE-corrected_*<0.05.

## Discussion

As hypothesized, the present study revealed a significant relationship between media multitasking and brain structure variations: Individuals who reported higher amounts of media multitasking had smaller gray matter density in the ACC. This association was significant at a stringent threshold (*p_FWE-corrected_<*0.05) and was independent of the Big Five personality trait differences. We discuss possible interpretations of our structural correlates in the light of recent evidence about ACC functions and MMI behavioral correlates.

The ACC serves as a crucial nexus of information processing pathways in the brain and has been implicated in sensorimotor, nociceptive, higher cognitive and emotional/motivational processes [22], [23]. Of these, we posit that our obtained ACC region is most likely linked with higher cognitive processes since media multitasking has been consistently associated with cognitive control performance [2], [10], [11], [24]. Furthermore, the ACC ROI exhibited significant functional connectivity with the DMN brain regions that were also typically linked with higher cognitive operations [25], [26].

In terms of cognitive processing, the ACC is generally thought to be involved in error or conflict detection [27], [28]. ACC activations are typically observed in tasks that concurrently activated incompatible responses i.e. the Stroop Task [29], [30], selective attention [31] and flanker task [32], [33]. Notably, ACC has been implicated in dual-task paradigms [34], [35] where an individual is faced with competing stimuli and responses associated with two or more tasks. Analogous to this, in media multitasking, individuals are confronted with distinct task demands associated with the multiple media types they are using simultaneously. As such, our obtained ROI could be implicated in dual-task related cognitive control functions. One critical caveat is that the abovementioned functions are normally attributed to the dorsal ACC as opposed to the rostral region where our ROI is situated [23], [32], [35], [36]. However, researchers have noted that this delineation is not absolute [23], [34], [37]. In particular, in support of our present interpretation, Dreher and colleagues [34] reported that the rostral ACC is uniquely involved in conflict detection in the context of dual-tasking.

Our main finding indicated that heavier media multitaskers had smaller ACC volumes. To elucidate possible behavioral implications of reduced ACC volumes in heavy multitaskers, we examined behavioral studies linking MMI and cognitive control. A landmark study by Ophir et al. [2] first revealed the relationship between increased media multitasking activity and poorer cognitive control. They engaged participants in a range of cognitive control tasks such as the Stroop task, task-switching, distractor filtering and the n-back tasks. In the face of distractors, heavy multitaskers (relative to lighter multitaskers) were slower in detecting changes in visual patterns, more susceptible to false recollections of the distractors during a memory task, and were slower in task switching. The authors suggested that heavy multitaskers were less able to volitionally restrain their attention only to task relevant information. Lui and Wong [24] provided further evidence that heavier multitaskers were worse at inhibiting task-irrelevant stimuli and consequently were able to perform better in multisensory integration tasks. A subsequent study [11] showed that heavy multitaskers performed worse on the Operation Span Task (OSPAN) which was highly similar to a dual-task paradigm since participants were required to concurrently solve math problems and memorize presented letters. Heavy multitaskers also reported more failures of attention in everyday life [38]. However, one recent study by Alzahabi and Becker [10] reported contrary findings: heavier multitaskers were not worse at dual-task performance and were better at task-switching. They were also unable to replicate Ophir et al.’s findings despite using identical tasks. The authors noted that their sample was primarily female and this might have resulted in their discrepant findings. They highlighted the importance of longitudinal studies to reveal robust relationships between MMI and cognitive control.

In summary, existing MMI literature generally suggests that individuals who engage in heavier media-multitasking show poorer cognitive control abilities. Our present findings extend this literature by linking heavier media-multitasking activity with smaller volumes in the ACC: a brain region that is implicated in cognitive control based on converging neuroimaging evidence. We emphasize, however, that more work is required to establish the relationship between ACC structure and cognitive control abilities. Studies of patients with ACC lesions have yielded very mixed perspectives about the necessity of ACC in its implicated cognitive functions [39], [40], [41].

There is also a possibility that our obtained ACC region is involved in emotional/motivational processes since it is situated in the rostral ACC that is typically linked with motivation and emotion processing [23]. Reduced ACC volumes have been often implicated in disorders involving aberrant emotional-motivational processing such as obsessive-compulsive disorder [42], post-traumatic stress disorder [43], depression [44] and drug and non-drug related addictions [45], [46]. Based on this perspective, it is plausible that heavier media multitaskers, with reduced ACC volumes, might be less disposed in emotional and motivational regulation. Indeed, higher MMI scores are found to correlate with increased neuroticism, sensation-seeking and impulsivity [3], [11] and negative socio-emotional outcomes [4]. Interestingly, the pattern of brain structural differences obtained in the present study was similar to the neural correlates of Internet addiction (IA). Individuals with IA, defined simply as pathological overuse of the Internet or computers, were found to have decreased gray and white matter densities in the ACC [46], [47], [48]. There could be a possibility that the two constructs, media multitasking and IA, were overlapping: the MMI provided a measure of how much people used multiple devices at once and this could be related to IA which implicates an excessive use of computers and internet.

An important limitation to the present work is that our results are obtained from a cross-sectional study on the relationship between media multitasking behavior and brain structure. As such, the direction of causality between them cannot be determined. Although it is conceivable that individuals with smaller ACC are more susceptible to multitasking due to weaker ability in cognitive control or socio-emotional regulation, it is equally plausible that higher levels of exposure to multitasking situations lead to structural changes in the ACC. A longitudinal study is required to unambiguously determine the direction of causation. Our current findings open a way to such research by providing an empirical link between media multitasking activity and structural differences in the ACC. One other caveat is that the present findings might not extend beyond our studied population that is relatively highly educated and well exposed to technology. Indeed media consumption patterns could be highly influenced by demographic factors [1]. As such, future studies should examine the role of demographic factors such as education and socio-economic status in moderating the relationship between media multitasking, cognitive performance and brain structures.

In conclusion, individuals who engaged in more media multitasking activity had smaller gray matter volumes in the ACC. This could also possibly explain the poorer cognitive control performance and negative socio-emotional outcomes associated with increased media-multitasking. While the cross-sectional nature of our study does not allow us to specify the direction of causality, our results brought to light novel associations between individual media multitasking behaviors and ACC structure differences.
